# The characteristics of gut microbiome changes in tuberculosis patients and latent tuberculosis infection in Xinjiang

**DOI:** 10.3389/fcimb.2026.1705360

**Published:** 2026-01-28

**Authors:** Yue Wang, Rukeyamu Abudushalamu, Xiaoming Peng, Kadierya Nasier, Zihao Teng, Yuwei Wang, Yuxue Chang

**Affiliations:** 1 Xinjiang Production and Construction Corps Center for Disease Control and Prevention, Urumqi, Xinjiang, China; 2 Xinjiang Uygur Autonomous Region Infectious Diseases Hospital, Urumqi, Xinjiang, China; 3 Xinjiang Uygur Autonomous Region Mental Health Center, Urumqi, Xinjiang, China; 4 Xinjiang Uygur Autonomous Region Hospital of Traditional Chinese Medicine, Urumqi, Xinjiang, China; 5 School of Public Health, Xinjiang Medical University, Urumqi, Xinjiang, China; 6 State Key Laboratory of Pathogenesis, Prevention and Treatment of High Incidence Diseases in Central Asia, Urumqi, Xinjiang, China

**Keywords:** 16S rRNA, diversity, gut microbiota, latent tuberculosis infection (LTBI), tuberculosis

## Abstract

**Objective:**

In our earlier research, the gut microbiota profiles of Uygur populations in Xinjiang infected with *Mycobacterium tuberculosis* (*Mtb*) characterized. As the Han and Uygur ethnic groups represent the predominant demographics in Xinjiang, this follow-up study focuses on identifying characteristic gut microbial alterations in Han patients with active tuberculosis (TB) and those with latent tuberculosis infection (LTBI). The findings are expected to support tailored strategies for the regional prevention and control of tuberculosis.

**Methods:**

A total of 51 cases of TB, 35 cases of LTBI and 51 healthy controls (HC) were recruited from the Infectious Disease Hospital of Xinjiang Uygur Autonomous Region. Fecal samples were collected and underwent 16S rRNA gene sequencing.

**Results:**

The gut microbiota α diversity was significantly lower in the TB group compared to the LTBI and HC groups, with significant β diversity differences observed among all three groups. At the phylum level, Firmicutes was the most abundant in all groups. The most abundant genera in the TB, LTBI, and HC groups were *Phocaeicola*, *Escherichia*, and *Bifidobacterium*, respectively. Lefse analysis revealed that pro-inflammatory and opportunistic pathogenic genera were enriched in the TB group, whereas butyrate-producing and immune-modulating genera dominated the LTBI group. PICRUSt2 analysis identified only five differential metabolic pathways between the TB and HC groups, among which *Clostridium* showed the strongest positive correlation with PWY-6876 (*R* = 0.79, *P* < 0.01).

**Conclusions:**

This study clarified the diversity, microbial species composition profiles, and metabolic pathways of the gut microbiota in the Han population with different TB states in the Xinjiang region of China. These findings may provide a certain theoretical basis and reference for the precise prevention and control of TB in Xinjiang.

## 1 Introduction

Tuberculosis (TB) is a long-standing disease caused by the bacterium *Mycobacterium tuberculosis* (*Mtb*). It poses a serious threat to human life and health and is one of the public health issues of global concern. According to the “2024 Global Tuberculosis Report” released by the World Health Organization, it was stated that: it is estimated that there will be 10.8 million new cases of TB worldwide in 2023, with an incidence of 134 per 100,000. The global incidence rate of TB has been on a downward trend during the period from 2010 to 2020, with an average annual decline of 2%. The disruption caused by the COVID-19 pandemic halted this positive trend, resulting in an estimated 4.6% rise in the global incidence of tuberculosis in 2023 compared to 2020. Among the 30 countries with high TB burdens, the estimated number of TB cases in China ranks third (accounting for 6.8% of the global total) (Organization W.H, 2024). Among Chinese regions, Xinjiang has a disproportionately high TB burden (Zhang et al., 2023), according to a spatiotemporal cluster analysis, the changes in incidence and mortality rates were also ranked among the top in the country (Guo et al., 2024). Furthermore, the global population with LTBI is approximately 2 billion. In China, about 550 million people are infected, making it the country with the highest burden of LTBI (Cui et al., 2020). This cohort has a 5–10% lifetime probability of progressing to active TB (Colangeli et al., 2020). Therefore, the LTBI population represents a large pool of potential patients (Chee et al., 2018).

This persistent disease burden arises from complex socioeconomic factors and host-pathogen interactions. Meanwhile, the gut microbiota participates in host physiology via metabolic crosstalk and immunomodulation, thereby serving as a pivotal regulator of systemic immune responses (Wang et al., 2023).The immunoregulatory role of the gut microbiota has been proven to be of crucial importance in the host’s response to TB, and involves the regulation of a series of dynamic changes such as *Mtb* infection, progression from LTBI to active disease, TB drug resistance, and the occurrence of co-infections (Hong et al., 2016). The gut microbiota modulates the host immune response to *Mycobacterium tuberculosis* (*Mtb*) through several key mechanisms: (1) direct modulation of immune cells. In innate immunity, microbial components such as LPS and peptidoglycan bind to pattern recognition receptors (including TLRs and NOD-like receptors) on host macrophages, dendritic cells, and intestinal epithelial cells, thereby modulating inflammatory responses; In adaptive immunity, the gut microbiota profoundly modulates the differentiation patterns of T cells, the primary cellular mediators of tuberculosis. (2) Metabolite-mediated immunoregulation. For instance, short-chain fatty acids (SCFAs) modulate macrophage polarization as well as B cell metabolism and antibody production. (3) The gut-lung axis alters the pulmonary microenvironment. This occurs primarily via systemic immune response activation by bacterial components and immune cell trafficking between mucosal sites. Conversely, perturbations in the lungs can reciprocally influence gut homeostasis (Liu et al., 2025).

The advancement of high-throughput sequencing technologies has made it possible to investigate the relationship between tuberculosis and gut microbiota. At present, to achieve the goal of eliminating TB, the focus of prevention and control should not only be on the infectious source population of active TB patients, but effective management of the LTBI population also needs to be regarded as an important approach (Fischer et al., 2025). We have previously investigated the changes in the gut microbiota of the Uyghur population with TB, LTBI and in HC in Xinjiang region (Wang et al., 2022a). Nonetheless, notable disparities exist in the gut microbiota profiles across distinct ethnic groups inhabiting the same geographical region (Zhang et al., 2015). Therefore, the present study aims to further clarify the changes in the gut microbiota among the Han population with different TB statuses in Xinjiang region. The study findings also intend to provide new ideas and a theoretical basis for more precise prevention and control of TB.

## 2 Materials and methods

### 2.1 Research subjects and sample collection

From July 2023 to July 2024, active pulmonary TB patients (TB group), latent tuberculosis infection carriers (LTBI group), and healthy controls (HC group) were recruited at the Infectious Diseases Hospital of Xinjiang Uygur Autonomous Region. This research was approved by the Ethics Committee of Xinjiang Medical University (XJYKDXR20220725019). All the research subjects signed an informed consent form prior to recruitment. The collection and processing of feces samples were as described in our previous paper (Wang et al., 2022a).

#### 2.1.1 Diagnostic criteria used for tuberculosis

The diagnosis and classification were conducted in accordance with “Diagnosis of Pulmonary Tuberculosis” (WS 288-2017) and “Classification of Tuberculosis” (WS 196-2017) issued by the National Health Commission of the People’s Republic of China.

#### 2.1.2 Inclusion and exclusion criteria for research subjects

##### 2.1.2.1 Inclusion criteria

Han ethnicity, aged 18 years or above, without taking antibiotics or other drugs that affect the intestinal flora for at least one month. The three groups of research participants were balanced in terms of average age and gender distributions, and they voluntarily participated in this study. (1) TB group: Patients diagnosed with new-onset active pulmonary TB, who had not started anti-TB treatment and had not taken anti-TB drugs. (2) LTBI group: No symptoms of TB, no history of TB, and no recent contact with TB patients. The TST test was positive, with an average diameter of the induration (PPD reaction) of ≥ 15 mm as the standard for TB infection. No preventive medication had been taken. (3) HC group: No symptoms of TB, no history of TB, no recent contact with TB patients, negative TST test result, and average diameter of the induration < 5 mm or no reaction.

##### 2.1.2.2 Exclusion criteria

None of the subjects had any other diseases, such as hepatitis B infection, HIV infection, tumors, autoimmune diseases, other lung diseases, and intestinal diseases.

### 2.2 DNA extraction and 16S rRNA gene amplification sequencing

As previously mentioned (Wang et al., 2022a), for the samples that had undergone pre-treatment, nucleic acids were extracted according to the kit instructions. The highly variable V3-V4 region of the 16S rRNA gene of microorganisms was amplified, and library construction was carried out using the TruSeq Nano DNA LT Library Prep Kit from Illumina and the samples were then analyzed.

### 2.3 Statistical analysis of baseline data

The data were statistically analyzed using SPSS 21.0. The count data are expressed as frequency and percentage, and the differences between the groups were analyzed using the χ^2^ test. Quantitative data are expressed as the mean ± standard deviation, and the differences between the groups were analyzed using analysis of variance. The significance level α was set at 0.05. A *P* value less than 0.05 indicated a statistically significant difference.

### 2.4 Bioinformatics analysis of 16S rRNA sequencing

As previously mentioned (Wang et al., 2022a), the 16SrRNA sequencing data were analyzed using the QIIME2 platform and the R software package (version 4.0.2). Quality control of sequencing data was achieved by using the number and length of high-quality fragments, as well as rarefaction curves. The taxonomic composition of the species is presented using a bar chart. Species diversity is illustrated and presented through relevant indicators of α and β diversity. Based on the species composition heatmap and the linear discriminant analysis effect size (Lefse) method, the different species were analyzed. Using phylogenetic investigation of communities by the reconstruction of unobserved states (PICRUSt2), we conducted an analysis to identify the differentially expressed metabolic pathways in the Metabolic Encyclopedia (MetaCyc) and Kyoto Encyclopedia of Genes and Genomes (KEGG) databases. The Spearman rank correlation method was employed to analyze the correlation between the genus-level biomarkers identified by Lefse analysis and the selected differential metabolic pathways. The correlation heatmap was employed to display the results. A difference was considered statistically significant when *P* < 0.05. According to the classification hierarchy proposed by Hebel (R H.J and J, 1979), a correlation coefficient *r*<0.2 is considered to have no correlation; 0.2 ≤ r < 0.5 indicates a weak correlation; 0.5 ≤ r < 0.8 represents a moderate correlation; and 0.8 ≤ r < 1.0 indicates a strong correlation. The sequence data of this study have been deposited in the GenBank Sequence Read Archive of NCBI under the accession code BioProject PRJNA1254675.

## 3 Results

### 3.1 Demographic characteristics

A total of 137 subjects were recruited for this study, including 51 cases of TB, 35 cases of LTBI and 51 HC. The average age of the three groups ranged from 54 to 56 years. The male-to-female ratio was 0.78:1. The subjects were comparable in terms of average age and gender (*P* > 0.05). Detailed patient information is shown in **Supplementary Table S1**.

### 3.2 Quality control of sequencing data

In this study, 137 samples were processed and ultimately resulted in 10,524,704 high-quality fragments. The average sequence length in the high-quality fragment was 417 bp, and the sequence range was from 230 to 442 bp. The rarefaction curves of the three groups of samples almost stabilized (**Supplementary Figure S1**).

### 3.3 Taxonomic composition analysis

All samples were averagely classified into one domain, 11 phyla, 14 classes, 27 orders, 47 families, and 100 genera (**Supplementary Table S2**).

**Figure 1:**
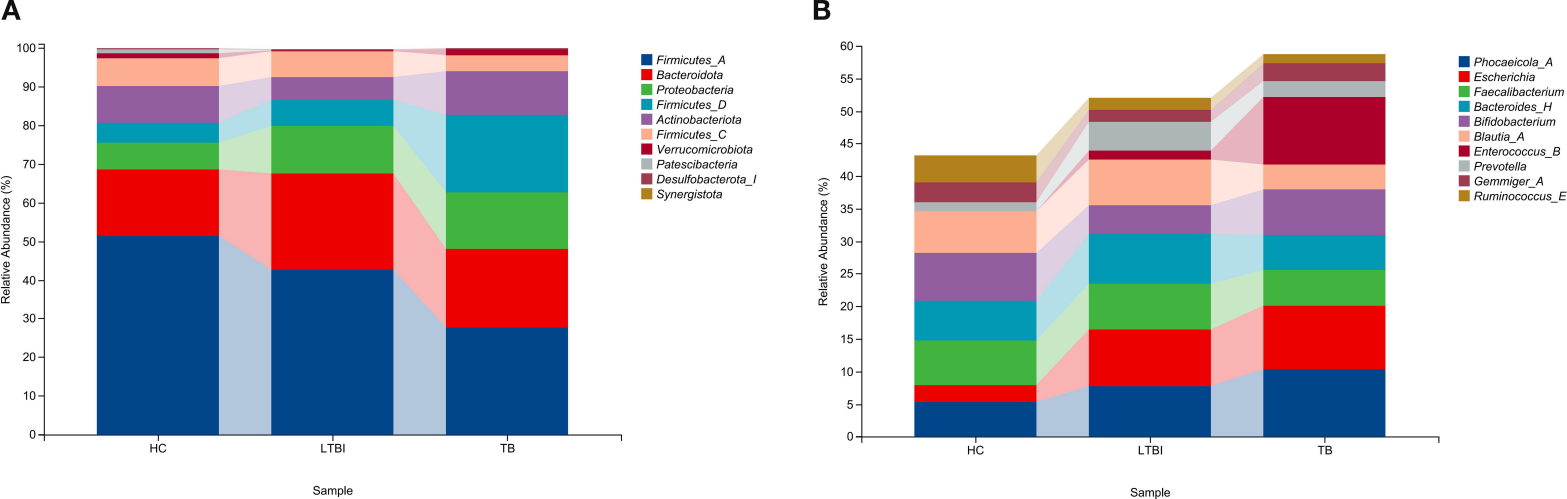
Histogram of the top 10 species composition of the three groups **(A)** at the phylum level. **(B)** at the genus level.

At the phylum level, the top ten phyla almost accounted for the entire species composition. *Firmicutes_A* had the highest abundance in the three groups. Its relative abundance in the TB group, LTBI group, and HC group was 27.91%, 42.72%, and 51.34%, respectively. At the genus level, the relative abundance of the top ten species in the TB group, the LTBI group and the HC group was 58.76%, 52.14% and 43.15%, respectively; In the three groups, the bacterial genus with the highest relative abundance was *Phocaeicola*_*A* (10.46%), *Escherichia* (8.64%) and *Bifidobacterium* (7.34%), respectively, the trend of changes in the other bacterial phyla (as shown in **Figure 1A**) and bacterial genera (as shown in **Figure 1B**) is also depicted.

### 3.4 Diversity analysis

**Figure 2:**
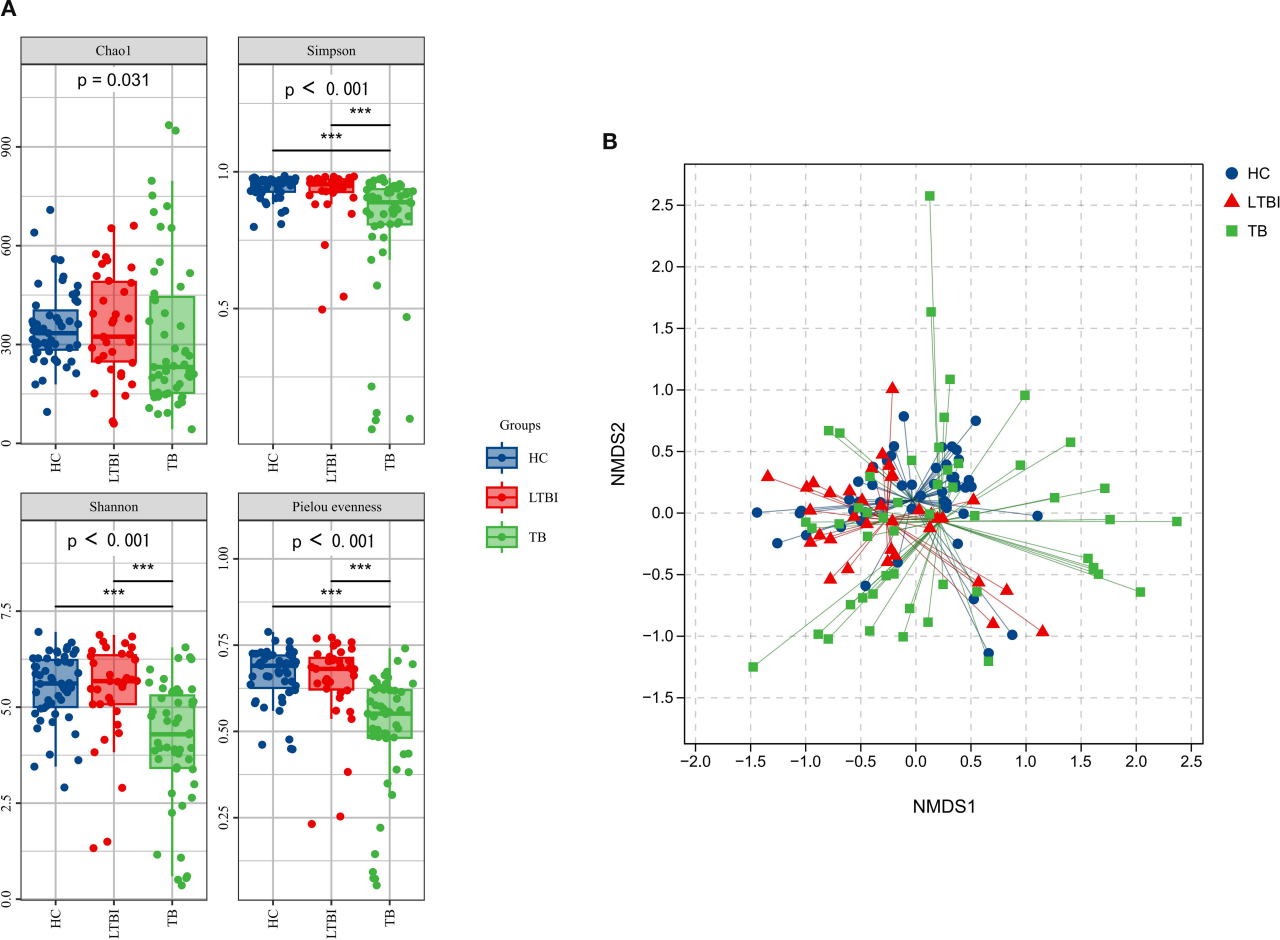
Alpha diversity and Beta diversity among the three groups. **(A)** Assessment of Alpha diversity among the three groups. The P-values of the overall difference between groups obtained, markers of difference significance levels obtained after pairwise comparison *P*<0.05;**, *P*<0.01;***, *P*<0.001. **(B)** Beta diversity among the three groups. NMDS represent beta diversity, measured by unweighted unifrac, the differences in the microbiome composition among groups were assessed by ANOSIM. Each point in the figure represents a sample, and points of the same color belong to the same group. The closer the distance between two points, the more similar the microbial composition of the two samples is. The scales on the X-axis and Y-axis represent relative distances.

Alpha diversity analysis showed significant difference among the three groups. The Simpson, Shannon, and Pielou’s evenness indices all indicated that the TB group showed differences compared to the LTBI group and the HC group (*P* < 0.001), and the diversity of the TB group decreased (**Figure 2A**).

Beta diversity analysis showed the three groups of samples at a specific distance scale (**Figure 2B**). The differences between the groups were greater than those within the groups (*P* = 0.001, *R* > 0). Further comparison revealed that there were differences between the TB group and the HC group, as well as between the LTBI group and the HC group (*P* < 0.05), but no differences existed between the TB group and the LTBI group.

### 3.5 Analysis of the composition of different species

The abundance of amplicon sequence variants (ASVs) was used to create a Venn diagram. The results are shown in **Supplementary Figure S2**. There were 10,874, 6,556, and 8,024 ASVs, respectively, in the TB group, LTBI group, and HC group; there were 9,332, 4,783, and 6,156 ASVs that were unique to each group. There was a total of 887 ASVs in the three groups.

**Figure 3:**
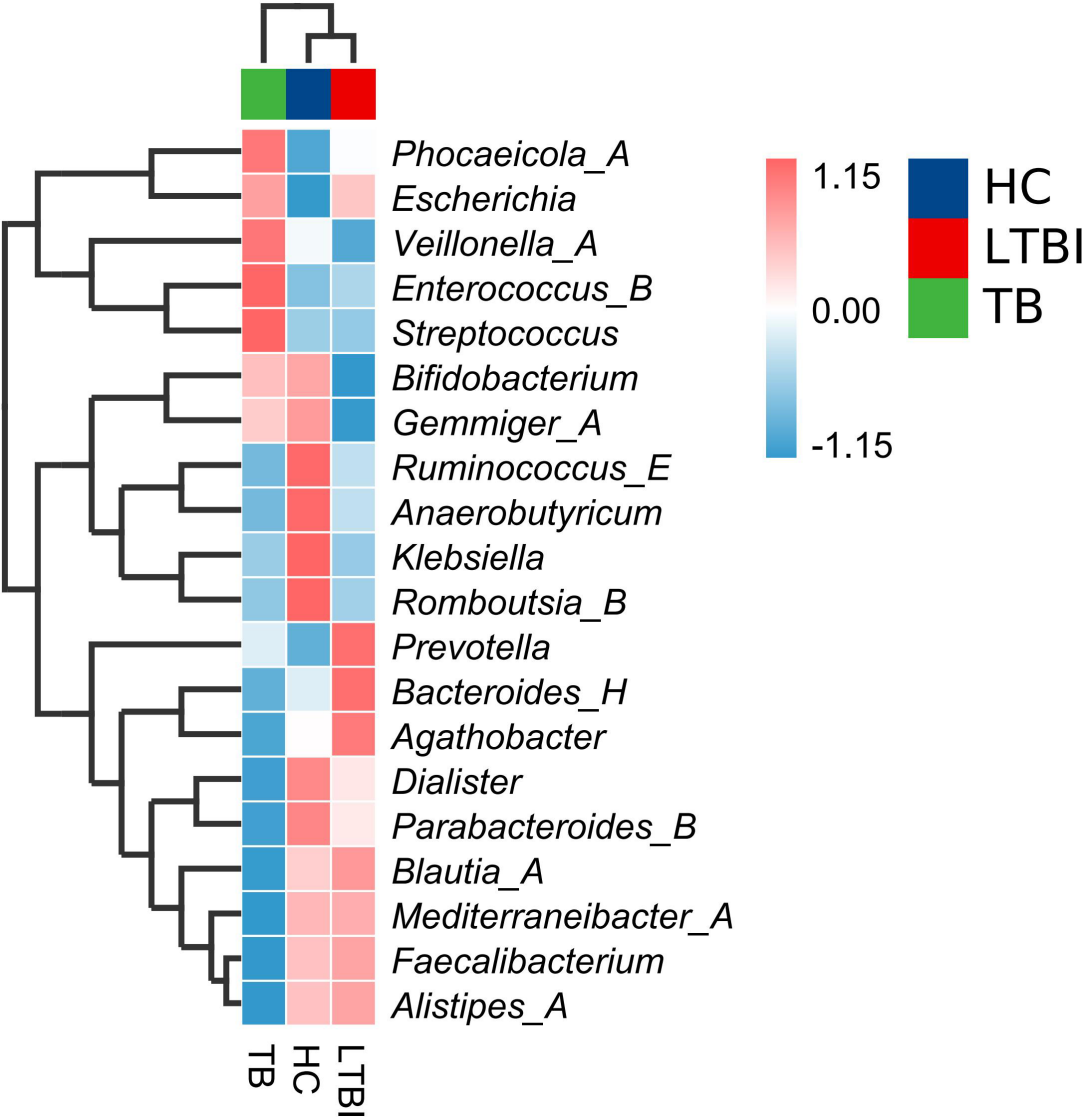
Cluster heat map of the top 20 species at the genus level of three groups. The gradient changes of colors (red and blue) in the heat map represent the changes in the relative abundance of genera. The values on the right corresponds to the color, which represents the abundance of the bacterial community in the sample. Each small square in the row represents a genus. The three small squares above represent the grouping situation. Green indicates the TB group, red indicates the LTBI group, and blue indicates the HC group.

To further compare the differences in species composition among the three groups, the trend of abundance distribution in each group is displayed. The “genus” level species with the top 20 average abundances were plotted in a visual heatmap, and cluster analysis was conducted for both the samples and the bacterial genera. The top 20 “genus” level species accounted for 68.73%. According to the distribution trend shown in the species composition heatmap, there were significant differences in the relative abundance of species among the three groups. The genera in the TB group and the LTBI group showed an opposite trend between the two groups, while the HC group was in the middle. The most abundant bacterial genera in the TB group were: *Streptococcus, Enterococcus_B*, and *Veillonella_A*; the most abundant bacterial genera in the LTBI group were: *Bacteroides_H*, *Prevotella*, and *Agathobacter*. The most abundant bacterial genera in the HC group were: *Klebsiella, Romboutsia_B*, and *Anaerobutyricum*. The abundance trends of the other bacterial genera are shown in **Figure 3**.

**Figure 4:**
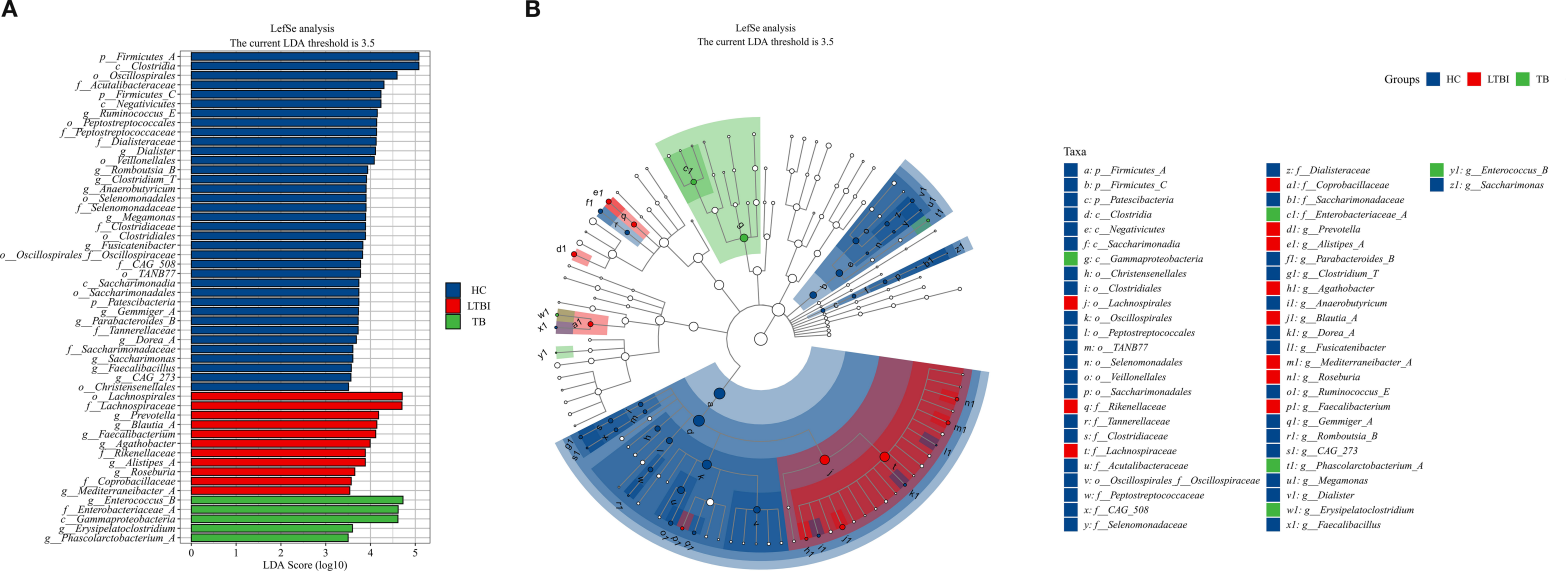
Lefse analysis for identifying microbial biomarkers **(A)** The barplot shows differentially abundant features and **(B)** The cladogram illustrates taxonomic relationships of differentially abundant features among three groups.

Based on the bar chart of the Linear discriminant analysis (LDA) values of different species (**Figure 4A**), three groups of species that were significantly enriched within the groups were identified; when the LDA threshold was set at 3.5, the 5 bacterial genera, namely *g_Enterococcus*, *f_Enterobacteriaceae, c_Gammaproteobacteria,g_Erysipelatoclostridium*, and *g_Phascolarctobacterium*, were regarded as biomarkers in the TB group; The 11 species, including *o_Lachnospirales*, *f_Lachnospiraceae* and *g_Prevotella, etc*, were identified as biomarkers in the LTBI group. While in the HC group, there were 36 species that were enriched, including *p_Firmicutes, c_Clostridia* and *o_Oscillospirales, etc.* From the cladogram (**Figure 4B**) of the species classification branch, the distribution of the taxonomic levels of the biomarkers in each group of samples can be seen.

### 3.6 Prediction analysis of differential metabolic pathways in the MetaCyc and KEGG databases

The 16SrRNA gene sequence was predicted in the MetaCyc database, and pairwise comparisons were performed between all groups. The results showed that there were four different metabolic pathways between the TB and HC groups. Among them, the pathways upregulated in the TB group compared to the HC group were PWY-6944, and the pathways downregulated in the TB group compared to the HC group were 3 in total, namely: P163-PWY, PWY-6876, and PWY-7374. Different metabolic pathways were not observed in the comparisons between the TB and LTBI groups, and between the LTBI and HC groups.

In addition, there was only one pathway that showed a difference and was downregulated in the TB group compared to the HC group in the KEGG database, which was ko00960. The primary metabolic pathway is metabolism, and the secondary pathway is biosynthesis of other secondary metabolites. No differences in metabolic pathways were found in the other two comparison groups.

### 3.7 Correlation analysis of indicator bacteria genera and differential metabolic pathways

**Figure 5:**
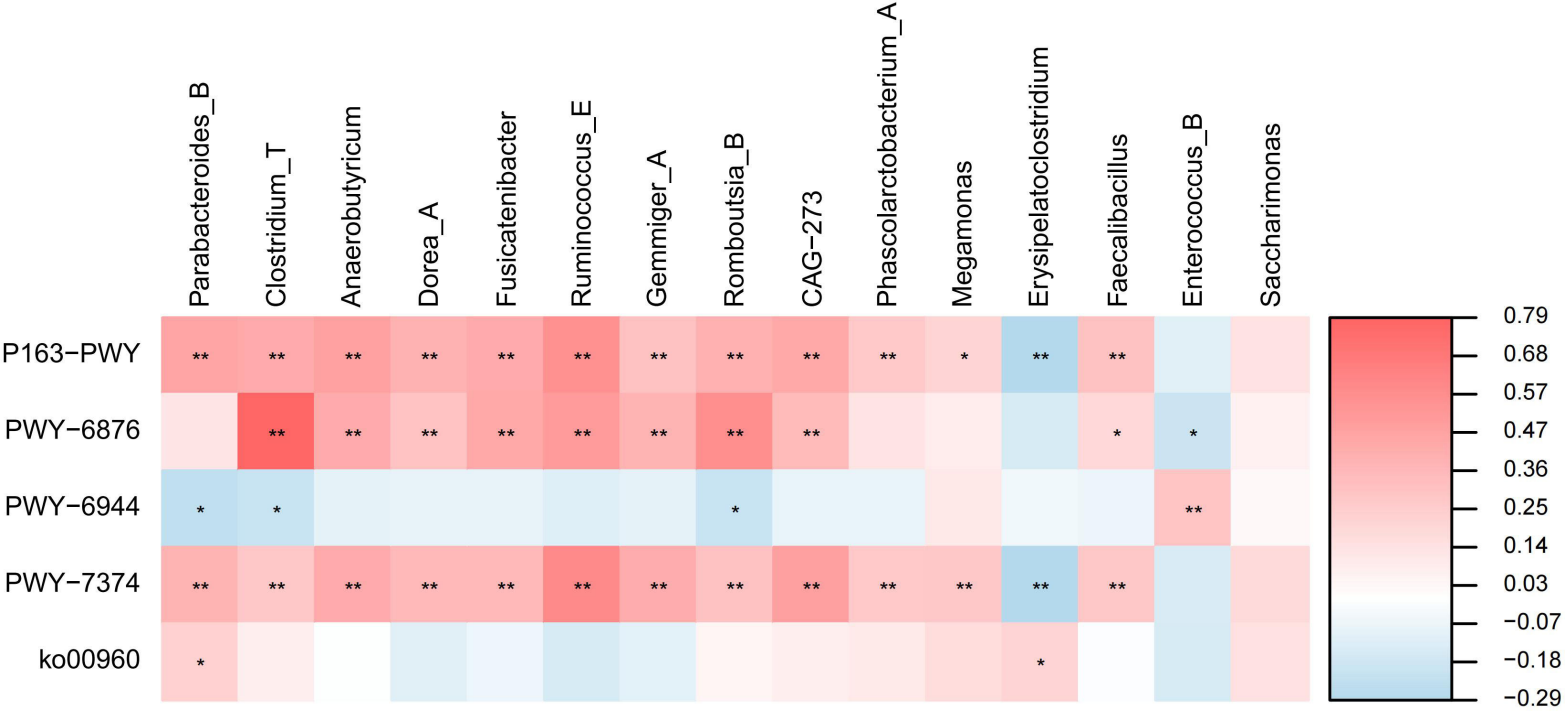
Correlation analysis between predictive metabolic pathways and microbial biomarkers in TB and HC groups. The depth of the color in the heat maps signifies the strength of the correlation: red represents a positive correlation, whereas blue indicates a negative correlation. *P < 0.05, **P < 0.01, ***P < 0.001.

In this study, only five different metabolic pathways were found to exist between the TB group and the HC group. The five differential metabolic pathways were correlated with the genus-level biomarkers identified in the Lefse analysis for both the TB group and the HC group, and the results were presented using a correlation heatmap (**Figure 5**). The strongest positive correlation was observed between *Clostridium_T* and PWY-6876 (*R* = 0.79, *P* < 0.01). *Erysipelatoclostridium* was associated with P163-PWY and PWY-7374 (*R* = -0.29, *P* < 0.01), which were the two groups with the strongest negative correlations in this study, but they were only weakly correlated.

## 4 Discussion

TB is a global public health issue and has attracted considerable attention. Existing studies have confirmed that there is a certain correlation between TB and the gut microbiota (Yu et al., 2023). Previously, in the Xinjiang region where TB is prevalent, we selected Uyghur individuals with TB and those with LTBI as research subjects to explore the change in characteristics of the intestinal microbiota. However, Zhang (Zhang et al., 2015) conducted a study on the gut microbiota involving 20 regions, 7 ethnic groups, and 314 healthy Chinese individuals aged 18–35 years. The study found that both regional and ethnic differences significantly affected the overall structure of the gut microbiota. The gut microbiota of individuals from the same ethnic group was similar, while those from different ethnic groups were different. Li (Li et al., 2016) compared the Tibetan population living at an altitude of 3600 meters with the Han population, and found that the intestinal microbial community of the Tibetan population exhibited a relatively higher abundance of *Prevotella*, while the Han population were rich in *Bacteroides*. Therefore, in this study, we aimed to further clarify the diversity and composition changes of the intestinal microbiota in the Han population in Xinjiang under different TB conditions, and to predict their potential functions, which could provide new reference data for the precise prevention and control of TB epidemics.

In terms of intestinal diversity, the α diversity in the TB group was the lowest among the three studied groups, lower than that of the LTBI group and the HC group. This is consistent with the results from the previous Uyghur population study (Wang et al., 2022a). In terms of β diversity, this study also used the same methods for testing, and notable disparities were observed among the three groups, with intergroup differences exceeding intragroup variations. Most existing studies have also shown that the α diversity in the TB group has decreased (Ding et al., 2022; Wang S. et al., 2022; Yang et al., 2022; Ye et al., 2022), and the β diversity showed differences in the TB group (Li et al., 2024). An exception, is the study by Luo, which indicated that diversity increased in both new-onset TB and recurrent TB cases, which might be due to the age differences among the subjects selected in the study (Luo et al., 2017).

In terms of the composition of the gut microbiome, the top-ranked phyla in this study were *Firmicutes* (57.19%), *Bacteroidota* (20.77%), *Proteobacteria* (11.34%), *Actinobacteriota* (8.93%), and *Verrucomicrobiota* (1.02%). In previous studies on the Uyghur population, the proportions were as follows: *Firmicutes* (72.08%), *Bacteroidota* (12.78%), *Actinobacteriota* (10.29%), *Proteobacteria* (4.00%), and *Verrucomicrobiota* (0.47%). In general, more than 90% of species belong to the *Bacteroidetes* and *Firmicutes* phyla, followed by the *Euryarchaeota* phylum and the *Actinobacteria* phylum. There are also relatively less abundant but very important phyla such as the *Papillomataceae* phylum, the *Fusobacteria* phylum, and the *Archaea* phylum (Lozupone et al., 2012). These findings are similar to our previous studies, but we can clearly conclude that the abundance of *Firmicutes* in Han subjects was lower than that in Uyghur subjects. The abundances of *Bacteroidota* and *Proteobacteria* were higher in Han subjects than in Uyghur subjects. Whether the Han population or the Uyghur population is considered, the abundance of *Firmicutes* in the TB group was lower than that in the LTBI group and the HC group. The abundances of *Proteobacteria, Actinobacteriota*, and *Verrucomicrobiota* were all higher in the TB group compared to the other two groups. *Bacteroidota* was also higher in the TB group than in the HC group. *Firmicutes* account for 64% of the intestinal flora in the general population. It has been widely recognized that its proportion decreases in TB patients (Piccioni et al., 2022). The decrease in *Firmicutes* is mainly due to the reduction of the two main bacterial families (*Lachnospiraceae* and *Ruminococcaceae*) that produce butyrate within this phylum (Liu et al., 2021). The reduction of butyrate will lead to an increase in pro-inflammatory responses, a decrease in antibacterial activity, and an increase in opportunistic pathogens due to damage to the epithelial barrier (Chen et al., 2019). *Bacteroidota* is the bacterial phylum that mainly produces acetic acid and butyric acid. Within this phylum, it mainly relies on the regulation of three bacterial genera, when the abundance of *Prevotella* decreases, the abundance of *Bacteroides* and *Parabacteroides* increases, and they jointly exert anti-inflammatory effects (Shi et al., 2021). In a previous report, the abundance of *Proteobacteria* was increased in the TB group, while in some studies, the abundance of *Actinobacteriota* showed the opposite trend (Ding et al., 2022; Wang S. et al., 2022; Li et al., 2024). These two bacterial phyla are predominantly opportunistic pathogens, and their presence is linked to mucosal destruction.

At the genus level, among the top ten genera, *Phocaeicola, Escherichia, Enterococcus* and *Ruminococcus* were found in this Han population, while they were not present in previous Uyghur research subjects. In addition, *Phocaeicola* and *Escherichia* were the two bacterial genera with the highest abundance in this study. The abundance of these two genera in the TB group was higher than that in the LTBI group and the HC group. The genus *Phocaeicola* belongs to the *Bacteroidetes* phylum and the *Bacteroides* family. *Escherichia* is a genus within the *Proteobacteria* phylum and the *Enterobacteriaceae* family. The abundance of *Enterococcus* in the TB group was significantly higher than that in the other two groups. In previous studies, they were also reported to increase in the TB group, and all are opportunistic pathogens that can cause colonization when the intestinal microbiota is imbalanced and the mucosa is damaged (Li et al., 2019; Shi et al., 2021; Ding et al., 2022; Li et al., 2024). The abundance of *Ruminococcus* was reduced in the TB group, which aligned with the findings of previous research (Hu et al., 2019).

The Lefse analysis was conducted to identify gut microbiota biomarkers. In the TB group, 5 biomarkers were identified at the specified threshold. Three were at the genus level: g_*Phascolarctobacterium_A*, g_*Erysipelatoclostridium*, and g_*Enterococcus_B*, which all belong to p_*Firmicutes.* The biomarker f:*Enterobacteriaceae* belongs to another type of biomarker, c:*Gammaproteobacteria*, which is also part of p:*Proteobacteria*. The predominant bacterial biomarkers in TB group were mostly pro-inflammatory and opportunistic pathogens (Achache et al., 2023; Chen et al., 2024). In the LTBI group, a total of 11 markers were identified, including seven at the genus level, three at the family level, and one at the order level. Mainly, the genera of the *Firmicutes* phylum mostly maintain the intestinal barrier and immune balance by producing butyric acid, and they are associated with chronic inflammatory diseases (Fusco et al., 2023; Wen et al., 2024). The *Bacteroidetes* phylum has diverse metabolic functions and may exhibit both pro-inflammatory and anti-inflammatory effects depending on the disease state (Halabitska et al., 2024; Xu et al., 2024). In addition, Luo et al. (2023) conducted LDA analysis on the gut microbiota of TB and LTBI populations and found that the LTBI group was rich in beneficial bacteria: *Romboutsia, Bifidobacterium* and *Lactobacillus*; while the TB group was rich in pro-inflammatory bacterial groups including *Ruminococcus gnavus, Streptococcus* and *Erysipelatoclostridium*.

In this study, the 16SrRNA gene sequence was predicted in the MetaCyc and KEGG databases. Only five pathways with differences were found in the TB group and the HC group. PWY-6944 (androstenedione degradation) is the only pathway among the 5 that was upregulated in the TB group. Androstenedione is a key intermediate of microbial steroid metabolism (Malaviya and Gomes, 2008), both *Mycobacterium tuberculosis* and *Mycobacterium tuberculosis* H37Rv possess this pathway (Nesbitt et al., 2010). The P163-PWY (L-lysine fermentation to acetate and butanoate) pathway is usually referred to as the lysine fermentation pathway, which is closely related to the production of short-chain fatty acids. Some species such as *Clostridium peptidivorans, Clostridium SB4*, and *Clostridium subterminale* can ferment L-lysine as the sole carbon and nitrogen source, producing butyrate, acetic acid, and ammonia (Han et al., 2024). PWY-6876 (isopropanol biosynthesis), the final product of this pathway, isopropanol, has broad-spectrum antibacterial and anti-inflammatory effects (Wang et al., 2022b), the enzymes catalyzing the steps of this pathway have been assembled from the following microorganisms: *Clostridium acetobutylicum, Clostridium acetobutylicum ATCC 824, Clostridium beijerinckii, Escherichia coli*, *etc* (Bermejo et al., 1998). In the current study, the positive correlation between *Clostridium* and the PWY-6876 pathway was the highest (*R* = 0.79, *P* < 0.01). The PWY-7374 (1,4-dihydroxy-6-naphthoate biosynthesis I) pathway is a futalosine pathway, which is usually associated with specific metabolic processes of biosynthetic pathways. The preliminary analysis of the sequenced genome indicates that this pathway may exist in multiple organisms. For example, in the gut microbiota of children diagnosed with autism spectrum disorder, this pathway indicates that it may be involved in the pathogenesis mechanism through immune regulation or metabolism (Lou et al., 2022). There are also multiple types of bacteria involved in this pathway, such as *Streptomyces coelicolor* and *Thermus thermophilus* (Hiratsuka et al., 2008). In our study, *Ruminococcus* was moderately correlated with the PWY-7374 pathway (*R* = 0.60, *P* < 0.01). However, the biological significance and mechanistic basis underlying this association require further investigation. The ko00960 (Tropane, piperidine and pyridine alkaloid biosynthesis), pathway starts with amino acids (ornithine, phenylalanine, lysine, aspartic acid), and mainly synthesizes biologically active nitrogen-containing compounds (alkaloids) (Kanehisa Laboratories, 2024). These compounds have ecological defense, anti-inflammatory, and antibacterial effects (Huang et al., 2019).

## 5 Conclusion

In this study, 16S rRNA gene sequencing was employed to further investigate the gut microbiota of Han populations with different TB statuses in Xinjiang region. The diversity, structural composition, and functional metabolism of the gut microbiota were characterized, and comparisons were made with previous studies on TB populations of Uyghur ethnicity. This research provides a theoretical reference for the implementation of precision prevention and control strategies against tuberculosis in Xinjiang.

## Data Availability

The datasets presented in this study can be found in online repositories. The names of the repository/repositories and accession number(s) can be found in the article/**Supplementary Material**.

## Supplementary material

Supplementary Table 1 Analysis of Demographic Characteristics.

Supplementary Table 2 Microbial taxonomic annotation at different levels.

Supplementary Figure 1 Rarefaction curves for the three groups of subjects.

Supplementary Figure 2 Venn diagram showing the shared and unique amplicon sequence variants (ASVs) of three groups.
