# Supplementary material for: The characteristics of gut microbiome changes in tuberculosis patients and latent tuberculosis infection in Xinjiang
Source: Front Cell Infect Microbiol. 2026 Jan 28;16:1705360. doi: 10.3389/fcimb.2026.1705360 (PMC12891185; doi:10.3389/fcimb.2026.1705360)
Supplement: Supplementary Table 1 — Analysis of Demographic Characteristics. [file Table1.docx]

**TableS1 Analysis of Demographic Characteristics**

| Variable | Category | PTB | LTBI | HC | ${}^{2}$/F  value | P  value |
| --- | --- | --- | --- | --- | --- | --- |
| Gender | male | 25（49.0%） | 15（42.9%） | 20（39.2%） | 1.013 | 0.603 |
|  | female | 26（51%） | 20（57.1%） | 31（60.8%） |  |  |
| Age（years） | average age | 54.03±16.78 | 56.45±16.26 | 55.7±15.53 | 0.235 | 0.791 |
|  | 18-44 | 9(30.0%) | 26(52.0%) | 16(28.1%) | 11.772 | **0.019** |
|  | 45-59 | 10(33.3%) | 13(26.0%) | 12(21.0%) |  |  |
|  | ≥60 | 11(36.7%) | 11(22.0%) | 29(50.9%) |  |  |
| BMI（kg/m²） | Underweight (< 18.5) | 4（7.8%） | 1（2.9%） | 1（2%） | 11.987 | 0.062 |
|  | Normal Weight (18.5–23.9) | 31（60.8%） | 19（54.3%） | 18（35.3%） |  |  |
|  | Overweight (24.0–27.9) | 12（23.5%） | 13（37.1%） | 25（49%） |  |  |
|  | Obesity (≥ 28.0) | 4（7.8%） | 2（5.7%） | 7（13.7%） |  |  |
| Smoke | yes | 12（23.5%） | 7（20%） | 8（15.7%） | 0.994 | 0.608 |
|  | no | 39（76.5%） | 28（80%） | 43（84.3%） |  |  |
| Drink | yes | 8（15.7%） | 3（8.6%） | 8（15.7%） | 1.104 | 0.576 |
|  | no | 43（84.3%） | 32（91.4%） | 43（84.3%） |  |  |
| Education | Primary school and below | 16（31.4%） | 10（28.6%） | 31（54.9%） | 2.247 | 0.069 |
|  | Junior middle school | 18（35.3%） | 10（28.6%） | 12（23.5%） |  |  |
|  | Senior high school and above | 17（33.3%） | 15（42.9%） | 11（21.6%） |  |  |
| Work Type | Physical Work | 15（29.4%） | 16（45.7%） | 13（25.5%） | 4.866 | 0.301 |
|  | Mental Work | 11（21.6%） | 7（20%） | 15（29.4%） |  |  |
|  | Retirement/unemployed | 25（49%） | 12（34.3%） | 23（45.1%） |  |  |
| Annual Household Income (10,000 yuan) | < 20,000 | 7（13.7%） | 2（5.7%） | 3（5.9%） | 3.74 | 0.442 |
|  | 20,000–50,000 | 20（39.2%） | 19（54.3%） | 26（51%） |  |  |
|  | > 50,000 | 24（47.1%） | 14（40%） | 22（43.1%） |  |  |
