# Supplementary figures and images for: The characteristics of gut microbiome changes in tuberculosis patients and latent tuberculosis infection in Xinjiang
Source: Front Cell Infect Microbiol. 2026 Jan 28;16:1705360. doi: 10.3389/fcimb.2026.1705360 (PMC12891185; doi:10.3389/fcimb.2026.1705360)

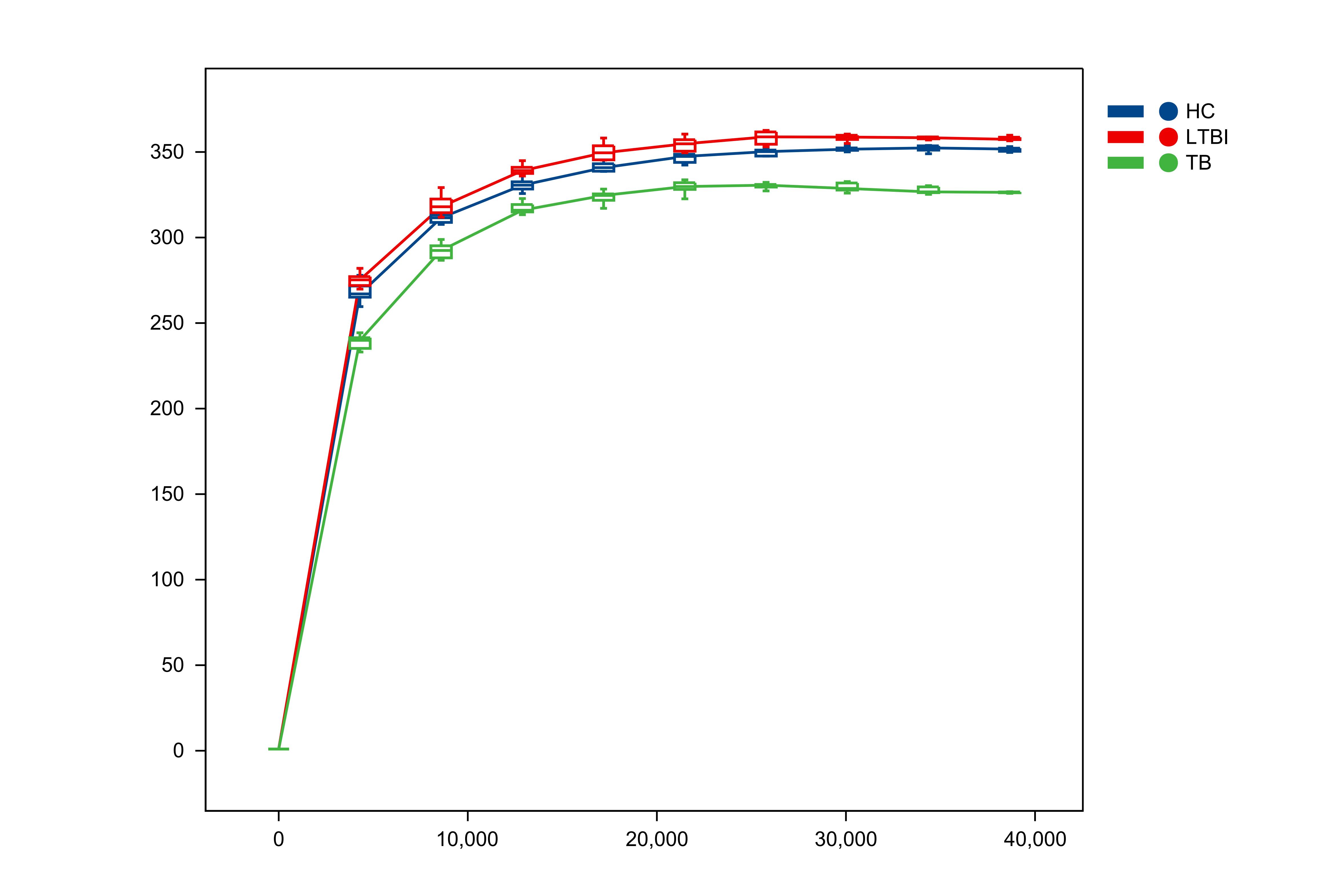

Supplement: Supplementary Figure 1 — Rarefaction curves for the three groups of subjects. [file Image1.jpeg]

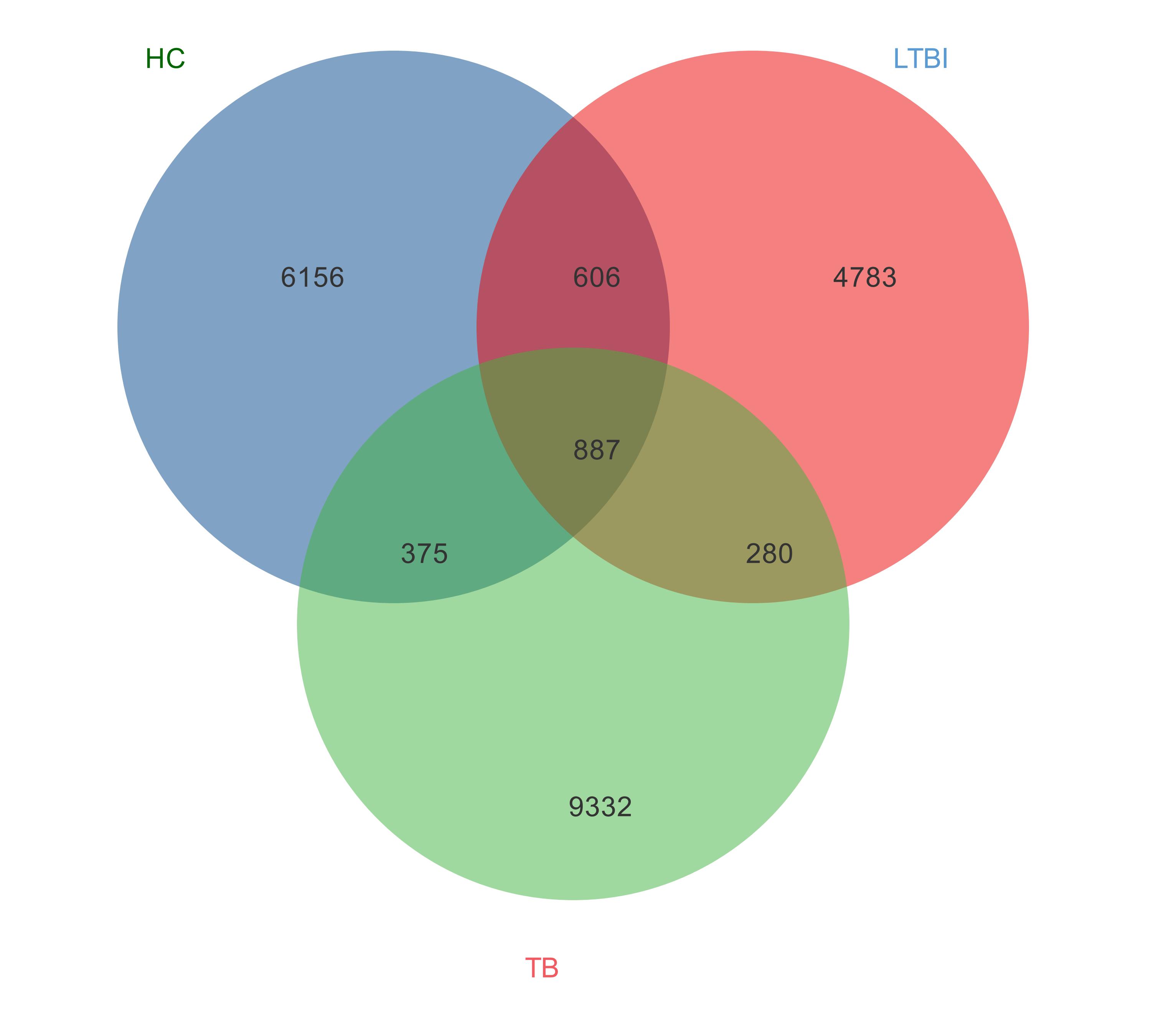

Supplement: Supplementary Figure 2 — Venn diagram showing the shared and unique amplicon sequence variants (ASVs) of three groups. [file Image2.jpeg]
